# Impact of COVID-19 pandemic on the neurosurgical practice in Egypt

**DOI:** 10.1186/s41984-022-00164-y

**Published:** 2022-06-07

**Authors:** Mohamed Nabil, Mohammed Dorrah, Asmaa Sharfeldin, Hassan Abaza

**Affiliations:** 1 Department of Neurosurgery, Menoufia University, Menoufia, Egypt; 2 Menoufia University Hospital, Menoufia, Egypt; 3 Department of Public Health and Community Medicine, Menoufia University, Menoufia, Egypt; 4 Department of Neurosurgery, Zagazig University, Sharkia, Egypt

**Keywords:** COVID-19, Neurosurgery, Lockdown, Pandemic

## Abstract

**Background:**

The COVID-19 pandemic and the subsequent lockdown have significantly altered many aspects of the health care services. We investigated the impact of the restrictive measures during the pandemic on the volume and spectrum of operated neurosurgical cases at two University hospitals in Egypt.

**Results:**

The total number of surgeries dropped during the lockdown (second quarter of the year 2020) by 38%, compared with the total number of surgeries in the first quarter of the same year, with an increase in the proportion of urgent surgeries to the total number of surgeries from 46 to 69% (*P* < 0.001), and a decrease in the proportion of elective surgeries from the total number of neurosurgeries from 54 to 31% (*P* < 0.001). Similar differences were noted in the volume and spectrum of surgeries in the second quarter of 2020, when compared to the same period of the preceding year (2019).

**Conclusions:**

The COVID-19 pandemic has significantly altered the nature and volume of neurosurgical practice. The overall number of surgeries showed a marked decline in the lockdown period; however, the numbers of urgent surgeries showed no significant difference under the lockdown.

## Introduction

The COVID-19 pandemic imposed an acute huge burden to most of the health care systems all over the world. The increasing need for ICU beds, mechanical ventilation and trained medical personnel deeply affected the course of surgical interventions in most of the hospitals worldwide. The neurosurgical practice has faced many difficulties since the beginning of the COVID-19 pandemic, forcing the neurosurgery departments to adopt an interim strategy of operating only the emergent and urgent cases and limiting or cancelling the elective cases [1].

On February 14, 2020, Egypt officially announced the first case of COVID-19 in Africa [2]. On March 8, Egypt announced the first death due to COVID-19 and on March 19, the Ministry of Health in Egypt announced that the total number of infected cases was 256 cases, including seven deaths [3]. On March 23, Egypt started to implement domestic restrictions (lockdown) to limit the spread of the virus. The Government declared a two-week curfew (from 7 p.m. to 6 a.m.) and announced the suspension of international flights and a closure of schools and universities till mid-April 2020. All cafes, malls, sporting clubs, and nightclubs were to be closed during the night-time curfew, only the food shops and pharmacies to be exempted [4]. On April 4, the cases surpassed 1000 [5]. Egypt has maintained the lockdown measures in action with a few serial reductions until the government declared lifting most of those measures on June 27, 2020. Starting from that date, the curfew ended, the international flights returned to be active, however the social distancing continued to be applied (Tables 1, 2). As of 25 May 2020, Egypt was among the five countries reporting the highest number of cases in Africa with a total of 17,265 cases [6].

**Table 1:** milestone dates of the COVID-19 spread in Egypt, with the corresponding cumulative numbers of cases and deaths

Date	Cases (cumulative)	Deaths (cumulative)
Feb 14, 2020	1	0
Mar 8, 2020	49	1
Mar 31, 2020	710	46
Apr 30, 2020	5537	392
May 31, 2020	24,985	959
Jun 30, 2020	68,311	2953

**Table 2:** The serial responses of the Egyptian government to the COVID crisis

Feb 14, 2020	Egypt announced the first case of COVID-19 in Africa
Mar 1, 2020	Egypt announced the second case of COVID-19
Mar 8, 2020	Egypt announced the first death due to COVID-19 (A 60-year-old German citizen)
Mar 9, 2020	The WHO confirms the presence of 56 COVID-19 cases in Egypt
Mar 23, 2020	Egypt declares two-week curfew (from 7 p.m. to 6 a.m.) and announces the suspension of international flights and a closure of schools and universities till mid-April 2020
Apr 4, 2020	COVID-19 cases in Egypt surpass 1000
Apr 8, 2020	Extension of curfew (from 8 p.m. to 6 a.m.) and international flights’ suspension until April 23
Apr 23, 2020	Extension of curfew through the month of Ramadan (ended in late May) with banning of all religious gatherings and ceremonies
May 19, 2020	Egypt indefinitely extends suspension of international flights
May 28, 2020	Shortening of the night-time curfew to start from 8:00 p.m. to 6:00 a.m.
June 14, 2020	Shortening of the night-time curfew to start from 8:00 p.m. to 4:00 a.m.
June 27, 2020	Egypt Lifts Most COVID-19 Restrictions and ends the night-time curfew

In this study, we analyse the impact of the COVID-19 pandemic and the related lockdown measures on the frequency of different neurosurgical interventions at two Egyptian University hospitals.

## Methods

This study was conducted at Menoufia and Zagazig University Hospitals, Egypt. We analyzed the pooled clinical data and operative notes of all patients operated by the Neurosurgery service of the two hospitals during the first halves of the years 2020 and 2019. The data were collected from the prospectively prepared electronic and paper records. The main study outcome was to detect the impact of the restrictive measures applied during the COVID-19 pandemic on the rates of different neurosurgical interventions.

Neurosurgical operations were described and analyzed according to their type. Then, they were subsequently classified according to time of surgery (degree of urgency) into three main types: 1- Emergent: this includes the surgeries performed immediately after the admission of the patient in order to save his/her life and/or neurological functions, 2- Urgent: includes surgeries performed in less than 24 h after the admission of the patient, 3- Elective: Surgeries that are carried out in the scheduled operative lists of the working days. Emergent and urgent surgeries were merged under one category to be more convenient to the aim of this study.

Urgent surgeries included traumatic brain injury (TBI), brain hemorrhage, acute hydrocephalus (CSF diversion and Shunt-related problems), spinal cord compression (spine fractures, infections, etc.) and peripheral nerve injuries. On the other hand, elective surgeries included degenerative spine disorders, spine deformity, spine tumors, brain space occupying lesions (SOL), endo-nasal skull base surgery, congenital malformations, peripheral nerve surgeries and cranioplasty.

Rate of different neurosurgical interventions during the first six months of the year 2020 was compared to the corresponding period of the preceding year (2019), as a control. For assessment of the impact of COVID-19 restrictive measures applied in March 2020 on the neurosurgical practice; rate of different neurosurgical interventions during the 1st and 2nd quarters of the year 2020 was compared (before and after applying the lockdown).

### Statistical analysis

Data were collected, tabulated and statistically analysed using an IBM compatible personal computer with Statistical Package for the Social Sciences (SPSS) version 23 (SPSS Inc. Released 2015. IBM SPSS statistics for windows, version 23.0, Armnok, NY: IBM Corp.) and Epi-calc 2000 program. Qualitative data were expressed as number and percentage (%). Z test was used for comparison of two proportions in two groups. *P* value > 0.05 was considered significant.

## Results

We found no significant difference between the rates of the different neurosurgical operations in the first quarter of the year 2020, compared to the same period of the year 2019 (*P* > 0.05) (Table 3). During the second quarter of the year 2020, the expected effect of the new restrictive hospital policies was evident in the form of a significant decline in the total number of surgeries by 38% when compared with the total number of surgeries in the first quarter of the same year (Table 4).

**Table 3:** Neurosurgical operations in the 1st three months of 2019 and 2020 (January, February and March)

	January 2019 (*n* = 284) *N* (%)	January 2020 (*n* = 276) *N* (%)	*P* value	February 2019 (*n* = 291) *N* (%)	February 2020 (*n* = 289) *N* (%)	*P* value	March 2019 (*n* = 298) *N* (%)	March 2020 (*n* = 251) *N* (%)	*P* value
*Type of surgery*									
Degenerative spinal disorders	98 (34.5)	86 (31.2)	0.5	94 (32.4)	88 (30.4)	0.7	96 (32.3)	76 (30.3)	0.7
Spinal fractures	17 (6)	18 (6.5)	0.9	19 (6.5)	22 (7.6)	0.73	17 (5.7)	20 (7.9)	0.4
Spinal infections	0	1 (0.4)	0.99	0	3 (1.1)	0.2	0	1 (0.4)	0.9
Spinal deformity	1 (0.4)	2 (0.7)	0.98	1 (0.3)	1 (0.3)	0.5	1 (0.3)	2 (0.8)	0.88
Spinal tumor	4 (1.4)	5 (1.8)	0.97	5 (1.7)	3 (1.1)	0.7	4 (1.3)	5 (2)	0.8
Brain SOL	31 (10.9)	26 (9.4)	0.7	32 (11)	34 (11.8)	0.9	28 (9.4)	22 (8.8)	0.9
Traumatic brain injury (TBI)	46 (16.1)	50 (18.1)	0.6	49 (16.8)	52 (18)	0.8	53 (17.8)	57 (22.7)	0.2
Brain hemorrhage	23 (8.1)	19 (6.9)	0.7	24 (8.3)	28 (9.7)	0.6	27 (9.1)	25 (9.9)	0.8
Endonasal skull base surgery	4 (1.4)	3 (1.1)	0.97	3 (1.03)	2 (0.7)	0.99	1 (0.3)	1 (0.4)	0.6
CSF diversion	19 (6.7)	21 (7.6)	0.79	19 (6.5)	20 (6.9)	0.98	27 (9.1)	19 (7.6)	0.6
Shunt-related problems	5 (1.8)	8 (2.9)	0.5	11 (3.8)	7 (2.4)	0.5	11 (3.7)	2 (0.8)	0.1
Congenital malformations	8 (2.8)	5 (1.8)	0.6	5 (1.72)	5 (1.73)	0.8	5 (1.7)	4 (1.6)	0.8
Peripheral nerve injury repair	1 (0.4)	5 (1.8)	0.2	1 (0.3)	3 (1.1)	0.6	2 (0.7)	0	0.6
Other Peripheral nerve lesions	27 (9.5)	27 (9.8)	0.9	28 (9.6)	18 (6.2)	0.2	26 (8.7)	17 (6.8)	0.5
Cranioplasty	0	0	–	0	3 (1.04)	0.2	0	0	–
*Time of surgery*									
Urgent	110 (38.7)	122 (44.2)	0.2	122 (41.9)	133 (46)	0.4	134 (44.9)	121 (48.2)	0.5
Elective	174 (61.3)	154 (55.8)	0.2	169 (58.1)	156 (54)	0.4	164 (55.1)	130 (51.8)	0.5

*SOL* space occupying lesion

**Table 4:** Neurosurgical operations in the 2nd 3 months of 2019 and 2020 (April, May and June)

	April 2019 (*n* = 350) *N* (%)	April 2020 (*n* = 180) *N* (%)	*P* value	May 2019 (*n* = 292) *N* (%)	May 2020 (*n* = 161) *N* (%)	*P* value	June 2019 (*n* = 293) *N* (%)	June 2020 (*n* = 167) *N* (%)	*P* value
*Type of surgery*									
Degenerative spinal disorders	149 (42.5)	28 (15.6)	< 0.001**	89 (30.5)	26 (16.1)	0.001**	84 (28.7)	23 (13.7)	< 0.001**
Spinal fractures	18 (5.1)	20 (11.1)	0.02*	18 (6.2)	22 (13.7)	0.01*	22 (7.6)	23 (13.8)	0.04*
Spinal infections	2 (0.6)	2 (1.1)	0.9	1 (0.3)	1 (0.6)	0.8	1 (0.3)	0	0.8
Spinal deformity	2 (0.6)	0	0.8	1 (0.3)	0	0.8	1 (0.3)	0	0.8
Spinal tumor	8 (2.3)	5 (2.8)	0.96	4 (1.4)	3 (1.9)	0.99	5 (1.7)	2 (1.2)	0.9
Brain SOL	27 (7.7)	21 (11.7)	0.2	29 (9.9)	17 (10.6)	0.96	26 (8.9)	16 (9.6)	0.9
Traumatic brain injury (TBI)	54 (15.4)	45 (25)	0.01*	59 (20.2)	41 (25.5)	0.2	61 (20.8)	65 (38.9)	< 0.001**
Brain hemorrhage	22 (6.3)	24 (13.3)	0.01*	21 (7.2)	19 (11.8)	0.1	22 (7.5)	14 (8.4)	0.9
Endonasal skull base surgery	2 (0.6)	0	0.8	3 (1.03)	1 (0.6)	0.9	2 (0.7)	1 (0.6)	0.6
CSF diversion	22 (6.3)	20 (11.1)	0.1	24 (8.2)	14 (8.7)	0.99	18 (6.1)	13 (7.8)	0.6
Shunt-related problems	12 (3.4)	9 (5)	0.5	10 (3.4)	11 (6.8)	0.2	8 (2.7)	6 (3.6)	0.8
Congenital malformations	4 (1.14)	2 (1.11)	0.7	4 (1.4)	4 (2.5)	0.6	7 (2.4)	4 (2.4)	0.8
Peripheral nerve injury repair	5 (1.4)	1 (0.6)	0.6	2 (0.7)	2 (1.2)	0.9	1 (0.3)	0	0.8
Other Peripheral nerve lesions	23 (6.6)	3 (1.6)	0.02*	27 (9.2)	0	< 0.001**	35 (12)	0	< 0.001**
Cranioplasty	0	0	–	0	0	–	0	0	–
*Time of surgery*									
Urgent	133 (38)	120 (66.7)	< 0.001**	133 (45.5)	108 (67.1)	< 0.001**	132 (45.1)	121 (72.5)	< 0.001**
Elective	217 (62)	60 (33.3)	< 0.001**	159 (54.5)	53 (32.9)	< 0.001**	161 (54.9)	46 (27.5)	< 0.001**

*Significant, **highly significant

**Fig. 1:**
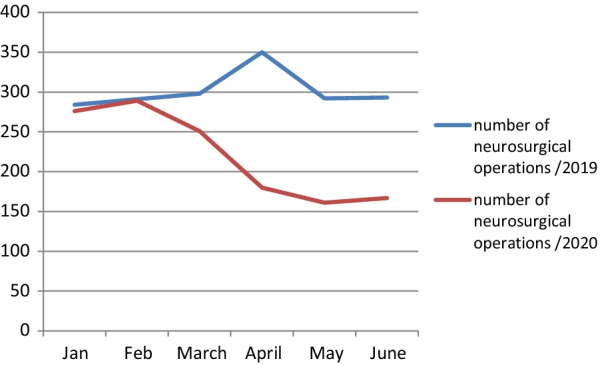
Number of neurosurgical operations in the 1st 6 months of 2019 and 2020

Also, we found an increase in the proportion of urgent surgeries to the total number of surgeries from 46 to 69% (*P* < 0.001), and a decrease in the proportion of elective surgeries from the total number of neurosurgeries from 54 to 31% (*P* < 0.001) (Table 4). Similar differences were noted in the volume and spectrum of surgeries in the second quarter of 2020, when compared to the same period of the preceding year (2019) (Table 5) (Fig. 1).

**Table 5:** Neurosurgical operations before and after the lockdown

	before COVID-19 (Jan. + Feb. + March) 2020 (*n* = 816) *N* (%)	after COVID-19 (april + May + June) 2020 (n = 508) *N* (%)	*P* value
*Type of surgery*			
Degenerative spinal disorders	250 (30.6)	77 (15.2)	< 0.001**
Spinal fractures	60 (7.4)	65 (12.8)	0.001**
Spinal infections	5 (0.6)	3 (0.6)	0.8
Spinal deformity	5 (0.6)	0	0.2
Spinal tumor	13 (1.6)	10 (1.9)	0.8
Brain SOL	82 (10.1)	54 (10.6)	0.8
Traumatic brain injury (TBI)	159 (19.5)	151 (29.7)	< 0.001**
Brain hemorrhage	72 (8.8)	57 (11.2)	0.2
Endonasal skull base surgery	6 (0.7)	2 (0.4)	0.7
CSF diversion	60 (7.4)	47 (9.3)	0.3
Shunt-related problems	17 (2.1)	26 (5.1)	0.004*
Congenital malformations	14 (1.7)	10 (1.9)	0.9
Peripheral nerve injury repair	8 (1)	3 (0.6)	0.7
Other Peripheral nerve lesions	62 (7.6)	3 (0.6)	< 0.001**
Cranioplasty	3 (0.4)	0	0.4
*Time of surgery*			
Urgent	376 (46.1)	349 (68.7)	< 0.001**
Elective	440 (53.9)	159 (31.3)	< 0.001**

*Significant (*P* value > 0.05), **highly significant (*P* value > 0.001)

It is important to note that there was no significant difference in the numbers of urgent surgeries performed during the second quarter of 2020, compared to the first quarter of 2020 and the first half of 2019. The significant difference was only in the percentage of urgent surgeries to the total number of surgeries (Table 5).

## Discussion

Based on our analysis, the first quarter of the year 2020 seemed very “normal” with operative rates very similar to the corresponding part of the year 2019. This could be attributed to the low number of PCR-confirmed cases in Egypt at that time, and to the possibly under-estimated burden of COVID-19 in Egypt. An earlier study by Tuite et al. claimed that the burden of infection in Egypt might be larger than reported. The study mentioned that as of March 6, 2020, Egypt has reported three cases of COVID-19; however, at least 14 cases have been exported from Egypt to four countries [7]. On 25 March 2020, the World Health Organization (WHO) concluded a COVID-19 technical support mission to Egypt. The mission reported that “Egypt is making substantial efforts to control COVID-19 outbreak, but more needs to be done”. The report mentioned that across the country, only 17 laboratories had the capacity to test cases, with more laboratories to be recruited [8].

Starting from March 2020, Egypt began to experience surges in the number of COVID-19 cases, which prompted the Egyptian government to adopt a plan of partial lockdown, with the application of a night-time curfew and a suspension of all international flights. All the hospitals across the country started to adopt new triage policies by minimizing or cancelling the elective surgeries and limiting the operative duties to the urgent and emergent cases, in addition to the strict detection measures applied to all patients before admission to the hospitals. Most people experienced major disruptions in their lifestyle, with the general population becoming less active, attending fewer social gatherings, and adhering to a quarantine regimen. Because of the severity and transmissibility of the disease, people were also hesitant to visit the ED of major hospitals for non-emergent issues because of concerns about catching or spreading the infection.

Based on that, we hypothesized that the lockdown period will cause a decline in the numbers of all types of neurosurgical operations. The elective surgeries would decrease because of the new hospital policies and the urgent surgeries also would decrease due to the expected decrease in traffic volumes, sport activities and assaults. Our data confirmed our expectations about the elective surgeries and showed a marked decline in their numbers after the application of the Anti-COVID-19 lockdown, however there was no significant decrease in the numbers of urgent surgeries after the lockdown; a finding that may reflect the inadequacy of the adopted lockdown strategy.

The COVID-related change in volume and the spectrum of operated cases has been reported by many reports lately, either because of the new hospital policies, or as a result of the lockdown [9–14]. Most of the available reports discussed cancellation of elective surgeries, the usage of telemedicine as an alternative to the outpatient clinics, and the increased usage of online lectures and virtual models to maintain the residents’ neurosurgical education [9, 10, 12–14].

### Limitations

We could not reach the full data of the years preceding the pandemic, and the comparison was done only between the pandemic year and the preceding one year. This was partly because of the absence of electronic databases and the difficulties to gather the data from the paper records. We also could not report the outcome of the patients whose elective surgeries were postponed, as many of them have shifted to other centers and private hospitals.

## Conclusions

The COVID-19 pandemic has significantly altered the nature and volume of neurosurgical practice. The overall number of surgeries showed a marked decline in the lockdown period; however, the numbers of urgent surgeries showed no significant difference under the lockdown.

## Data Availability

The data that support the findings of this study are available from Menoufia and Zagazig University hospitals, but restrictions apply to the availability of these data, which were used under license for the current study, and so are not publicly available. Data are however available from the authors upon reasonable request and with permission of Menoufia and Zagazig University hospitals.

## Abbreviations

COVID-19: Coronavirus Disease 2019
TBI: Traumatic brain injury
CSF: Cerebrospinal fluid
SOL: Space-occupying lesion
PCR: Polymerase chain reaction
ED: Emergency department
